# Sex-stratified piRNA expression analysis reveals shared functional impacts of perinatal lead (Pb) exposure in murine hearts

**DOI:** 10.1080/15592294.2025.2542879

**Published:** 2025-08-10

**Authors:** Kimberley E. Sala-Hamrick, Kai Wang, Bambarendage P. U. Perera, Maureen A. Sartor, Laurie K. Svoboda, Dana C. Dolinoy

**Affiliations:** a Department of Environmental Health Sciences, School of Public Health, University of Michigan, Ann Arbor, MI, USA; b Department of Computational Medicine and Bioinformatics, University of Michigan Medical School, Ann Arbor, MI, USA; c Department of Pharmacology, University of Michigan Medical School, Ann Arbor, MI, USA; d Department of Nutritional Sciences, School of Public Health, University of Michigan, Ann Arbor, MI, USA

**Keywords:** piRNA, Pb, heart, cardiac, toxicoepigenetics, sex differences, environmental epigenomics, developmental origins of health and disease (DOHaD)

## Abstract

The landscape of PIWI-interacting RNA (piRNA) expression in the heart is poorly understood, particularly regarding sex differences. Altered piRNA expression has been reported in cardiovascular disease (CVD), and although exposure to the metal lead (Pb) is strongly associated with CVD risk, no studies have investigated Pb’s effects on cardiac piRNAs. This study aimed to characterize piRNA expression in the murine heart and assess sex-specific effects of human-relevant maternal Pb exposure on adult offspring cardiac piRNA expression. piRNAs were identified from whole mouse hearts using sodium periodate exclusion of small RNA and subsequent sequencing. Control mice expressed 18,956 piRNAs in combined-sex analysis; sex-specific analyses revealed 9,231 piRNAs in female hearts and 5,972 piRNAs in male hearts. Genomic mapping showed 28–41% aligned to introns, while 12–28% mapped to exons. Comparing control and Pb-exposed hearts, we found more potential Pb-induced expression changes in females (847) compared to males (187) (p-value < 0.05 and |logFC| > 1). These piRNAs were significantly enriched near genes involved in biological processes related to heart function and CVD development, including mitochondrial function, energy metabolism, and cardiac muscle structure (FDR < 0.05). Overall, we characterized combined and sex-stratified piRNA expression in both control and Pb-exposed murine hearts. In addition to providing a foundation for sex-specific piRNA expression in the heart, these findings suggest a novel epigenetic mechanism by which developmental Pb exposure may impact CVD risk later in life. Future studies will link these sex-specific molecular changes to Pb-induced alterations in cardiac function.

## Introduction

Epigenetic modifications, which include DNA methylation, histone modifications, and non-coding RNA (ncRNA), contribute to changes in gene expression without altering the underlying genetic code itself [1]. PIWI-interacting RNA (piRNA) are a class of small non-coding RNA (smRNA) first coined in the early 2000s by a group of studies showing a novel distinct small RNA which is associated with PIWI proteins in both *Drosophila* and mammals [2–6]. piRNAs have since been shown to play important roles in maintaining genomic stability in germ cells by controlling DNA methylation of transposable elements and later to control transposable element expression through cleavage of mRNA targets (post-transcriptional gene silencing) [7–9]. piRNAs have also been shown to alter histone modifications leading to heterochromatin formation as well as to regulate posttranslational modifications of transcription factors [10]. piRNAs are longer in length than other micro RNAs, as germline piRNAs are roughly 21–35 nucleotides [11]. Primary piRNAs have a strong preference for a 5ʹ uridine signature, and secondary piRNAs have a bias for an adenine signature at position 10 [12,13]. piRNAs are also distinct from other forms of small non-coding RNA in that they have a 2’-O-methylation at their 3’ terminus which confers stability [14]. This 2’-O-methylation can be harnessed to select for piRNA compared to other small RNA through treatment with sodium periodate, which oxidizes all molecules terminating in 2′,3′-OH groups, preventing linker attachment and thus only allowing RNA with 2’-O-methylation to be sequenced [15]. This method has previously been shown to be effective in identifying somatic piRNA expression and is more rigorous than using size exclusion [15–17].

Although they are well characterized in the germline, little is known about piRNA function and sex-specificity in somatic tissues, including the heart. In the soma, piRNAs were first discovered to play important roles in stem cell function and in the development of multiple types of cancer [18–20]. More recent studies have found that piRNA pathways are also important in fully differentiated somatic tissues, particularly in the brain, where they have been implicated in neurodegenerative disease [21]. Our recent work has demonstrated that somatic piRNA (albeit shorter in length versus germline piRNA) and associated *Piwils* are expressed in the mouse soma, including the brain, liver, and kidney [15].

Some work has found altered piRNA expression in the context of cardiovascular disease (CVD) [22,23]. piRNAs have been shown to play roles in cardiac repair, differentiation, and pathologies [22–25]. They are expressed in models of cardiac repair (cardiospheres and cardiosphere-derived cells) and may be responsible for regenerative potential of cardiosphere progenitors [24]. It has been postulated that they also play critical roles in fine tuning gene expression during differentiation of embryonic stem cell-derived cardiomyocytes [25]. Disease-specific piRNAs have been implicated in both *in vivo* and *in vitro* rat models of cardiac hypertrophy, as well as in epidemiological studies [22,23]. Interestingly, in models of cardiac hypertrophy, piRNA expression was found to be increased *in vivo*, mapping to mRNA regions, and decreased *in vitro*, mapping to both mRNA and transposon (including LINE-1) regions [22]. This study also found specific piRNAs elevated in myocardial infarction patients [22]. One epidemiological study found large differences in expression (both up and downregulation) of piRNAs in heart failure patients [23]. piRNAs were also found to be differentially expressed in patients with chronic thromboembolic pulmonary hypertension (CTEPH) [26].

Importantly, there are prominent sex differences in manifestations, pathogenesis, and prognosis of CVD, but whether differential piRNA expression underlies these disparities is unclear [27,28]. piRNAs display a degree of sex-specific expression in the germline, with zebrafish models showing that over 20% of piRNAs have differential expression between ovaries and testes [29,30]. It is hypothesized that sex differences in piRNA expression arise from variations in transposable element activity due to differences in germ cell development [31]. Comparatively, little is known about sex-specific piRNA expression in the soma. Other ncRNA including micro RNAs (miRNAs) and long non-coding RNAs (lncRNAs) have been shown as sex-specific mediators or biomarkers of CVD, although this research is in its early stages [32].

The Developmental Origins of Health and Disease (DOHaD) paradigm states that environmental exposures during critical periods of life (e.g., periconception, gestation, infancy, adolescence) impact the onset of disease in later life, and this has been demonstrated in humans as well as animal models (including mice) [33]. Environmental pollutants are known to play roles in the incidence and progression of CVD. Lead (Pb) is a heavy metal and common environmental contaminant released from industrial sources or found in older homes or buildings, and Pb exposure is associated with adverse cardiovascular health outcomes [34]. However, mechanisms of Pb’s role in CVD are still being explored, and how sex differences influence this effect has yet to be characterized. Environmental exposure to Pb has been characterized to regulate gene expression through epigenetic modifications in both humans and mice [35–41]. One of the mechanisms by which Pb may contribute to the development of CVD is through altered regulation of the epigenome. Perinatal exposure to Pb has been shown to alter DNA methylation and gene expression in a sex-specific manner in mouse cardiac tissue, and these changes map to pathways important for cardiac development and disease progression [41–43]. Thus, given that piRNAs modulate gene expression through altering DNA methylation, it is important to assess whether Pb exposure impacts piRNA expression.

Overall, the aims of this project were to identify heart-specific piRNA in male and female mice and characterize longitudinal piRNA expression influenced by perinatal Pb exposure. This work may lead to the use of piRNA as a potential biomarker of disease and opens the door for the potential to use piRNA for interventions to treat CVD [44].

## Methods

### Mouse breeding experiments

Mice (*mus musculus*) used for this study have recently been described [45,46]. All mice for this study were housed at the ULAM (Unit for Laboratory Animal Medicine) of University of Michigan and maintained on regular 12–12 dark-light cycles at 70–73°F and 50% humidity. Animals were maintained on a phytoestrogen-free modified AIN-93 G diet (TD.95092, 7% Corn Oil Diet, Envigo, Indianapolis, IN, USA). All animals had access to food and drinking water *ad libitum* throughout the experiment and were housed in polycarbonate-free cages. Health checks were carried out daily by lab personnel and ULAM staff. Health checks consisted of a general assessment of appearances (fur coat intact and well-groomed, eyes clear, no signs of fight wounds) and behavior (mobility, nest building, etc.). In addition to checks by lab personnel, a designated animal handler from ULAM checked these cues on a daily basis, and a veterinarian assessed the health status of the mice at least once a week. At 5 months of age, all (both control and Pb exposed as outlined below) mice were euthanized with CO_2_ asphyxiation followed by a secondary method of euthanasia of bilateral pneumothorax procedure in accordance with rules and regulations set forth by the Institutional Animal Care and Use Committee (IACUC). This study protocol was approved by the University of Michigan IACUC protocol #PRO00009800. ARRIVE guidelines were adhered to for this study.

### Mouse exposure paradigm

**Figure 1.**
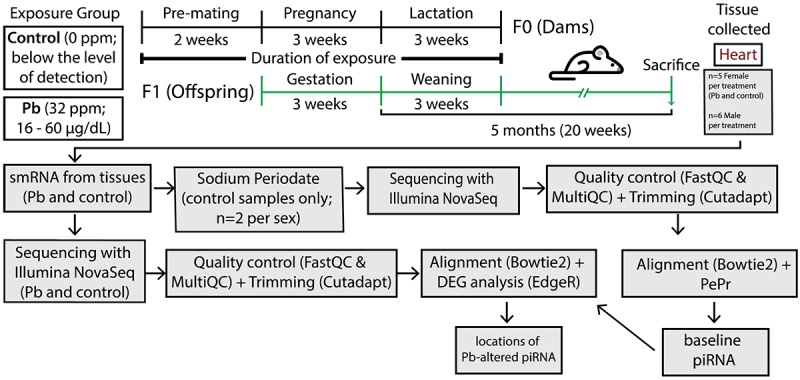
Experimental design for perinatal mouse Pb exposure and sequencing analysis pipeline. Pb exposure began with female mice two weeks prior to mating and continued through lactation until weaning at 3 weeks of age. After weaning, all offspring received Pb-free water until sacrifice at 5 months of age. Approximately 1–2 male and 1–2 female offspring per litter were sacrificed (*n* = 5 female and *n* = 6 male per exposure group). After collecting smRNA from whole heart tissues, sodium periodate treatment was performed on select control hearts (*n* = 2 per sex) to select for baseline piRNA, followed by sequencing on Illumina NovaSeq, quality control (FastQC and Multiqc) and trimming (cutadapt), alignment to the genome (Bowtie2), and PePr peak calling to distinguish peaks enriched in the sodium periodate treated samples (piRNA). Another set of smRNA (all collected samples; *n* = 5 female and *n* = 6 male per treatment) was sequenced, underwent quality control (FastQC and Multiqc) and trimming (cutadapt), alignment to the baseline piRNA generated by previous sequencing using STAR, EdgeR analysis to compare piRNA expression in control vs. Pb-exposed, and the resulting locations of piRNA alterations associated with Pb exposure was used in downstream analyses.

This perinatal Pb exposure study was done as part of a larger study under the National Institute of Environmental Health Sciences (NIEHS) Toxicant Exposures and Responses by Genomic and Epigenomic Regulators of Transcription (TaRGET II) Consortium [46]. Pb preparation and exposure were conducted exactly as outlined previously [39]. Virgin female wild-type *a/a* non-agouti mice (derived from a colony for the viable yellow agouti (*A*^vy^) strain) 93% identical to the C57BL/6 mice (6–8 weeks old) were randomly assigned to receive control or Pb via drinking water 2 weeks prior to mating, followed by mating with virgin a/a males of the same strain (7–9 weeks old; Figure 1) [45]. Pb II acetate trihydrate (32 ppm; Sigma-Aldrich, Milwaukee, WI, USA) was mixed with distilled drinking water, which results in a human-relevant maternal exposure in the 16–60 µg/dL range [39,47]. Pb exposure continued through mating (for both males and females), pregnancy (~3 weeks), and lactation until weaning at 3 weeks of age (Figure 1) [42]. After weaning, all offspring received Pb-free water until sacrifice at 5 months of age (Figure 1). Approximately 1–2 male and 1–2 female offspring per litter were sacrificed (*n* = 5–6 per sex per exposure; Figure 1). No formal sample size calculation was performed. Sample sizes were based on standard practice in the laboratory and were consistent with prior publications using similar methods [48]. Animal collection occurred between 1 and 3 pm, with collection order randomized daily [48]. As outlined in previous studies, one investigator administered treatment and was therefore aware of treatment group allocation, but all investigators completing subsequent molecular assays were blinded to the treatment group until it was analyzed during bioinformatic analysis [48]. All experiments were conducted according to experimental procedures delineated by the NIEHS TaRGET II Consortium [46,48]. Whole hearts from these mice were snapped frozen in liquid nitrogen and stored at −80°C at the time of sacrifice. The outcome of interest for this study was piRNA expression in the heart.

### Tissue lysis and smRNA extraction

Heart tissue was cryo-pulverized and small RNA (smRNA) extraction was performed using the AllPrep DNA/RNA Mini Kit (#80204 Qiagen, Aarhus, Denmark) and RNeasy MinElute Cleanup Kit (#74204, Qiagen, Aarhus, Denmark) according to the manufacturer’s instructions. The AllPrep kit selects for total RNA, which includes RNAs that are > 200 bp, while the RNeasy kit was used to further select for small RNAs that are < 200 bp. Sample concentration and quality were assessed using a nanodrop microvolume spectrophotometer (Thermo Fisher Scientific, Waltham, Massachusetts, USA).

### Sodium periodate exclusion to select for baseline piRNA assessment

Control heart smRNA samples (*n* = 2 per sex, not littermates per sex) were treated with sodium periodate to select for piRNA against other forms of smRNA. piRNAs are resistant to sodium periodate treatment due to the presence of a unique 3’ modification of 2’-O-methylation [15,16,18]. Sodium periodate treatment was performed as previously described [15]. Briefly, four separate reactions were set up, each with 400 ng of smRNA (27 µL) with the same amount of borate buffer added (8 µL; generated with 150 mM borax: #T29C533, Alfa Aesar, Ward Hill, Massachusetts, USA, and 150 mM boric acid: #SZBG1380V, Fluka Analytics, Buchs, Switzerland, and adjusted to a pH 8.6 using sodium hydroxide: #A4782902, Thermo Fisher Scientific, Waltham, Massachusetts, USA), but three reactions with sodium periodate added (5 µL; #BCBS5360V, Sigma Aldrich, Milwaukee, WI, USA) and the other water (5 µL), and then samples are incubated for 30 minutes. This was followed by an ethanol precipitation, resuspension of smRNA in water, and combination of the three sodium periodate treated smRNA samples into one.

### Sequencing of smRNA

smRNA sequencing was performed in two separate batches for this study. For the first, periodate treated (*n* = 2 per sex) and non-treated samples (*n* = 2 per sex) were sequenced (total *n* = 8) at the University of Michigan’s Advanced Genomics Core (AGC). Before sequencing, quality control and library preparation were performed by the AGC using TapeStation (RNA ScreenTape #5067–5576 and RNA ScreenTape Sample Buffer #5067–5577, Agilent Technologies, Santa Clara, CA, USA; analysis software 4.1) and SMARTer smRNA-seq kit (#635031, Takara, San Jose, CA, USA). Purification was performed to remove excess adapter present in samples. Sequencing was performed on an Illumina NovaSeq S1 flowcell (200 cycle) ensuring ~ 38 million reads per sample. For our second batch of sequencing, using non-periodate treated samples but including smRNA from hearts of mice that had been treated with Pb (*n* = 5 female per treatment and 6 male per treatment for a total of 22 samples), we followed the same sample preparation procedures as were done for the first batch, and sequenced on an Illumina NovaSeq S4 300 cycle.

### Computational analysis: read processing

Reads were analyzed according to a piRNA analysis pipeline established previously [15]. First, raw data quality was assessed using FastQC (v0.11.8) and MultiQC (v1.8). Next, processing of the reads was performed through adapter trimming, size selection (10–45 bp), and quality trimming. After this, Bowtie2 (v2.3.5.1) was used to align reads to the genome, using option -k 1000 –score-min ‘C,0,-1’ to find the perfect alignments for reads with 1000 mapping locations.

### Computational analysis: heart-specific piRNA selection

Control samples were used to create baseline maps of piRNA present in the mouse heart. PePr peak calling software (version 1.1.21 with parameters – shiftsize 0 – windowsize 20 – threshold 1E–3 – peaktype sharp) was utilized for pairwise comparison of the filtered alignments from sodium periodate treated and control samples to identify regions overrepresented in periodate treated RNA reads [49]. This step ensures that regions identified are those that are significantly more resistant to the sodium periodate [49].

Peaks shorter than 20 nucleotides or greater than 45 nucleotides were excluded from subsequent analyses. The website https://csbg.cnb.csic.es/BioinfoGP/venny.html and package eulerr in R were used to create Venn diagrams of expression in different analyses (combined, female, and male) of baseline piRNAs. Genomic annotations were carried out using the Bioconductor package Annotatr (v1.24.0) in R. Full information on genomic annotations can be found here: https://bioconductor.org/packages/devel/bioc/vignettes/annotatr/inst/doc/annotatr-vignette.html.

R package valr (v0.6.8) was used to perform the repeat annotation against the repeat annotation downloaded from the UCSC Genome Browser (https://hgdownload.soe.ucsc.edu/goldenPath/mm10/bigZips/file: mm10.fa.out.gz), and subsequently the categories were condensed into themes (i.e. LTRs, LINEs and SINEs) and other RNA types were omitted from results. The results for both genomic and repeat annotations were plotted using ggplot2.

### Computational analysis: piRNA expression upon Pb exposure

To study the effects of Pb exposure, we performed smRNA sequencing (no sodium periodate) and compared results to the baseline piRNA map from control hearts. This alignment was performed using STAR and featureCounts (v2.0.3). Next, differential expression of piRNAs in Pb vs. control treated samples was assessed using generalized linear modeling with EdgeR (v3.40.2) package in R. These results were filtered for significance by p-value (none were significant by FDR) and a |logFC| > 1 and then were plotted using ggplot2 R package. Venn diagram creation, genomic annotations and repeat analysis were performed as described above.

For Gene Ontology analysis of the piRNAs potentially altered by Pb exposure (p-value < 0.05 and |logFC| > 1), the Bioconductor package chipenrich (v2.22.0) was used to perform the gene set enrichment testing, in which piRNA-enriched peaks were assigned to target genes using the nearest transcription start site (TSS) (locusdef = ‘nearest_tss’), and enrichment was evaluated using the chipenrich logistic regression model, which adjusts for the number of peaks per gene. The input was piRNAs potentially differentially expressed in Pb treated samples (as determined by edgeR), and GO genesets GOBP (Biological Processes), GOCC (Cellular Compartment), and GOMF (Molecular Function) were used. The results were reduced so that similar processes were narrowed down, filtered for gene sets less than 500 genes, FDR < 0.05, and plotted using ggplot2.

### qRT-PCR of potential mRNA targets

RNA was extracted from the cyro-pulverized mouse heart samples (*n* = 4 per sex per treatment) described above using the AllPrep DNA/RNA Mini Kit (#80204 Qiagen, Aarhus, Denmark). 1 μg of total RNA was reverse transcribed into cDNA using the iScript cDNA

Synthesis Kit (#1708890 BioRad, Hercules, California). mRNA expression of mouse genes *Atf2* and *Apbb1* was quantified using the CFX Real-Time PCR using iTaq Universal SYBR Green Supermix (primers: qMmuCID0016589, qMmuCED0039791, SYBR: #1725271, BioRad, Hercules, California) according to manufacturer’s protocols with all samples run in triplicate. mRNA expression was compared using the same method on the same plate to amplification of and two reference genes (*Gapdh* and *B-actin*), which were IDT primers with sequences: *Gapdh*: forward primer: TGACCTCAACTACATGGTCTACA and reverse primer: CTTCCCATTCTCGGCCTTG and *B-actin*: forward primer: GTGACGTTGACATCCGTAAAGA and reverse primer: GCCGGACTCATCGTACTCC (Integrated DNA Technologies, Coralville, Iowa). Primer efficiency was confirmed using cDNA serial dilutions. All qRT-PCR reactions were carried out for 40 cycles under standard PCR conditions. ΔCq values were calculated by subtracting the Cq triplicate average of a target sample from that of the geometric mean of the reference genes. The relative difference in gene expression was then calculated using 2^−∆Cq^ and graphed using GraphPad Prism, where unpaired t-tests were used to determine statistical differences between control and treatment groups.

## Results

### Identification of sex-stratified murine heart-specific piRNA using sodium periodate exclusion

To characterize sex-specific cardiac expression of piRNA, we treated smRNA from control mouse hearts (*n* = 4, 2 per sex) with sodium periodate to enrich for piRNA over other smRNA. We used a gold standard sodium periodate method to select for smRNA containing the 2’-O-methylation, sequenced all periodate treated and untreated samples, then utilized the PePr peak calling software to identify the relative enrichment of piRNA in treated vs. untreated samples, and further selected for piRNAs based on their length (20–45 nucleotides) [15,16].

**Figure 2.**
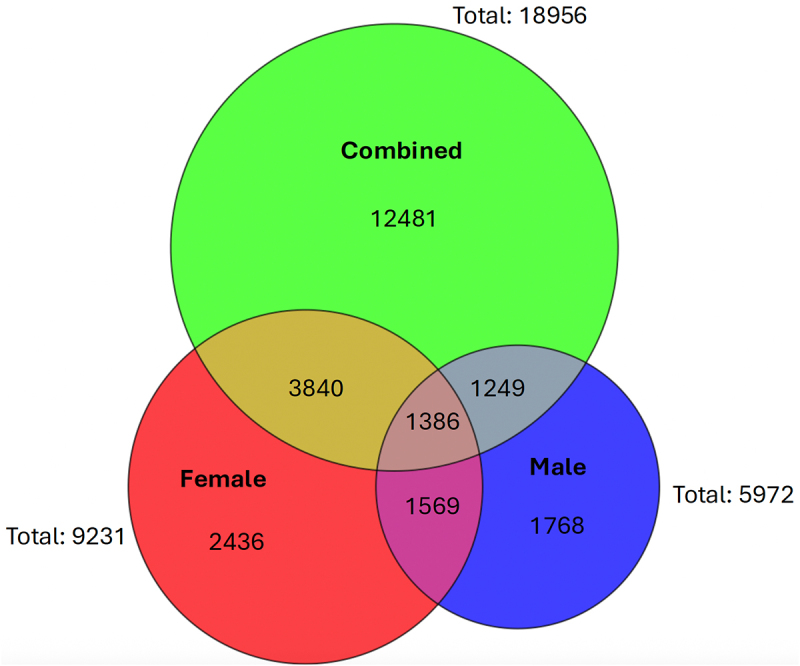
Combined and sex-stratified baseline heart piRNA expression. The Venn diagram shows overlaps in expression of 20–45 nucleotide piRnas discovered through PePr analysis (Table 1) from combined (green), female (red), and male (blue) analyses.

We conducted three assessments of the piRNA sequencing results: combined sex analysis and stratified by sex (female and male). The average number of total million reads was 36.9 in females and 15.9 in males (Table 1). The alignment rates were between 65–75% for all three assessments, and approximately half of reads mapped to multiple loci for all three analyses (Table 1). A total of 32,035 enriched regions (representing candidate piRNAs) were present in the heart (for combined sex), with 18,956 of these at transcript lengths between 20–45 nucleotides (Table 1). In the sex stratified analyses, there were 16,756 female derived enriched regions (9,231 meeting length criteria) and 11,408 male derived enriched regions (5,972 meeting length criteria) (Table 1). Assessing overlaps in expression of piRNA sequences that met length criteria shown in the Venn Diagram in Figure 2, 1,386 were found to share expression in all three analyses, with 2,436 unique to only females and 1,768 unique to only males.

**Table 1.** Summary of piRNA expression in control hearts identified by sodium periodate exclusion, including combined and sex-stratified piRNA assessment.

	Average total reads (millions)	Alignment rate (%)	Mapped to multiple loci (%)	All enriched peaks	piRNA 20 to 45 nucleotides
Combined	26.38	69.82	54.95	32035	18956
Female	36.89	73.36	58.51	16756	9231
Male	15.87	66.29	51.38	11408	5972

### Genomic annotations of cardiac piRNAs in adult mice reveal heart-specific locations

**Figure 3.**
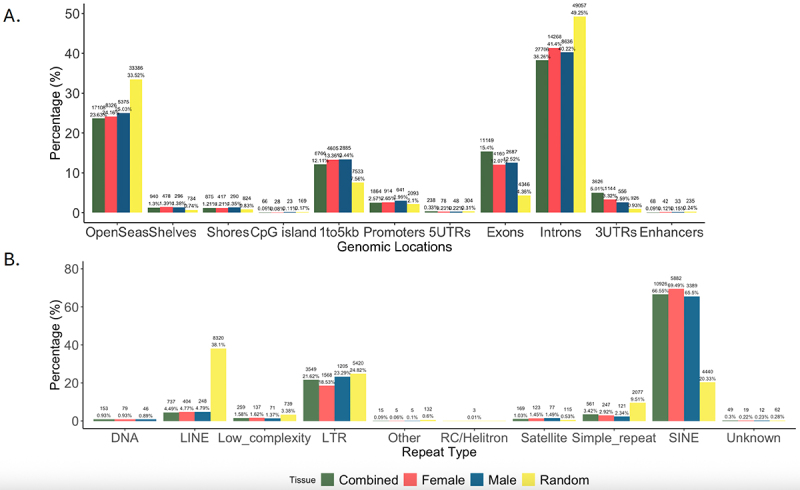
Genomic annotations and repetitive regions for expression of baseline heart piRNAs. Genomic annotations were generated for the regions from which 20–45 nucleotide piRNAs described in table 1 were derived for combined sex analysis (green), female (red), male (blue), and a random region of the mm10 genome (yellow) using annotatr in R (a) The locations annotated included OpenSeas (interCGI), CpG shelves, CpG Shores, CpG Islands, 1to5kb (upstream of transcription start site), promoters, 5’ UTRs, exons, introns, 3’UTRs, and enhancers. Annotations of repetitive regions were also generated for these piRNAs (b) The repeat annotation was performed by using the R package valr against the repeat annotation downloaded from the UCSC genome browser. Types of repetitive regions were condensed into the following categories: DNA, LINE, Low_complexity, LTR, other, RC/Helitron, satellite, Simple_repeat, SINE, and unknown. Tables S1 (gene annotations) and S2 (repeat analysis) contain the frequency and proportions labelled on the graph for each analysis.

We annotated the heart-specific piRNAs to the mouse version mm10 genome and found that the 18,956 piRNAs combined across sexes mapped most frequently to introns, open sea areas, and exons (Figure 3(a)). Interestingly, this pattern diverged from what would be expected in a random area of the mm10 genome (shown in yellow in Figure 3(a)). This annotation revealed higher proportions of piRNA (for combined sexes, females, and males) mapping to exons (12–15%) and lower proportion of piRNA mapping to introns (38–41%) compared to the expected proportion of exons for a random region of the mm10 genome (4% exonic regions and 49% intronic regions) (Figure 3(a)). Sex differences were not seen in genomic annotations.

Analysis of piRNA mapping to known repetitive regions in the genome revealed a lower proportion of piRNAs in long interspersed nuclear elements (LINEs) and similar proportions of piRNAs mapping to long terminal repeat (LTR) retrotransposons compared to the proportion of those repetitive regions in a random region of the genome (LINEs: 4–5% in analysis compared to 38% in a random region; LTRs 18–23% in analysis vs. 25% in a random region) (Figure 3(b)). The proportion of piRNAs mapping to SINEs in all three analyses was much higher (65–69% compared to 20% in a random region). Tables S1 (gene annotations) and S2 (repeat analysis) contain the frequency and proportions labelled on the graphs for Figure 3(a,b), respectively.

### Heart-specific piRNA expression patterns upon perinatal Pb exposure

**Figure 4.**
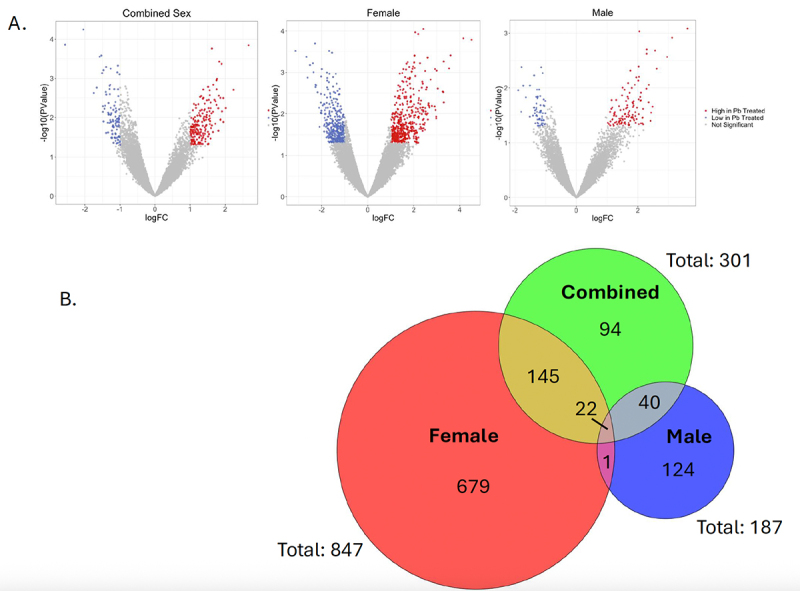
Potential changes in expression of heart piRNAs from mice developmentally exposed to Pb. After mapping piRnas to the 32,035 peaks in control hearts, edgeR was used to assess for changes in expression with Pb exposure compared to control samples in combined sexes, female, and male hearts (*p*-value < 0.05). Highlighted regions in blue and red correspond to downregulated and upregulated piRNAs described in Table 2 (log Fold change < −1 or > 1; (a) Respectively. The Venn diagram shows overlaps in these piRNAs from combined (green), female (red), and male (blue) analyses (b).

smRNA from hearts of adult control mice (*n* = 5 females and *n* = 6 males) and mice that had been exposed to Pb during gestation and lactation (*n* = 5 females and *n* = 6 males), all at 5 months of age, were aligned to the 32,035 sequences identified in the combined sex PePr analysis. We included all 32,035 sequences, rather than just those that met the size selection criteria, to ensure that we captured any potential piRNAs that might be present in the broader range of piRNA lengths. While none of the piRNA in Pb-exposed samples were found to be significantly different compared to controls (FDR < 0.05), we identified hundreds of potential piRNA expression changes with |log(fold change (FC))| > 1 and p-value < 0.05 (Table 2 and Figure 4). Using the aforementioned criteria, more potential piRNA expression changes with Pb exposure were identified in the female samples (847) than in males (187) or the combined analysis (301), and only 23 overlapping piRNAs were found between males and females; Table 2 and Figure 4(b). For the combined analysis, of the 301 potentially changed piRNAs found with exposure, 199 (66.11%) had a greater than two-fold change increase and 102 (33.89%) had a greater than two-fold change decrease (Table 2). Of the 847 piRNAs changed with Pb exposure in females, 486 (57.38%) had a greater than two-fold change increase and 361 (42.62%) had a greater than two-fold change decrease (Table 2). Of the 187 piRNAs potentially affected by Pb exposure in males, 122 (65.24%) had a greater than two-fold change increase and 65 (34.76%) had a greater than two-fold change decrease (Table 2). Overall, the proportion of potentially upregulated versus downregulated piRNAs was relatively consistent across all three groups, with a higher percentage showing increased expression following Pb exposure.

**Table 2.** piRNA changes (p-value < 0.05 and log Fold change (FC) > 1 or < −1) associated with perinatal Pb exposure in the mouse heart.

	Combined	Female	Male
logFC > 1	199 (66.11%)	486 (57.38%)	122 (65.24%)
logFC < −1	102 (33.89%)	361 (42.62%)	65 (34.76%)
Total	301	847	187

### Genomic annotations of heart-specific piRNAs sensitive to Pb exposure

**Figure 5.**
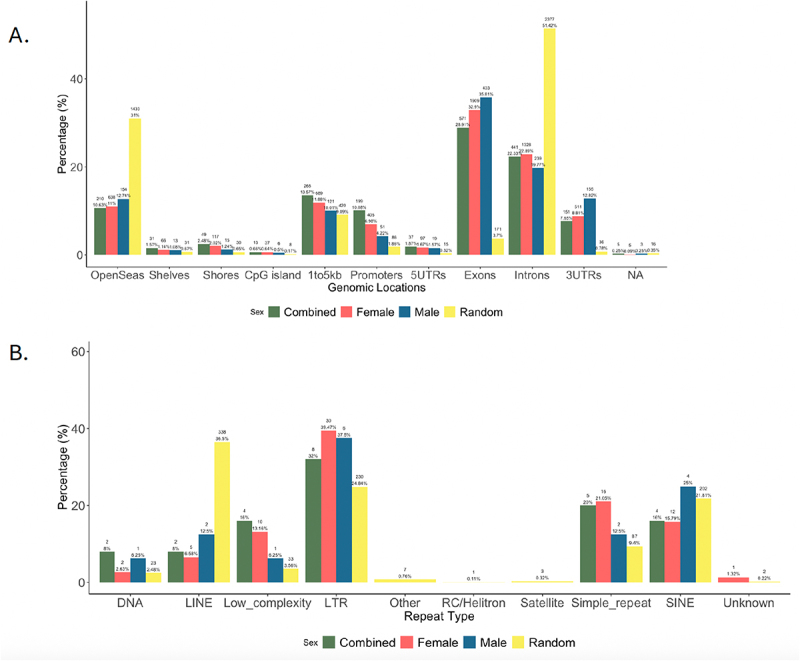
Genomic annotations and repetitive regions for expression of heart piRNAs with potential changes in expression in mice developmentally exposed to Pb. Genomic annotations were generated for regions potential changes in expression of piRnas by Pb exposure (p-value < 0.05 and logFC > 1 or < −1) were derived from combined sex analysis (green), female (red), male (blue), and a random region of the genome (yellow) using annotatr in R comparing to the mm10 mouse genome (a) The locations annotated included OpenSeas (interCGI), CpG shelves, CpG Shores, CpG Islands, 1to5kb (upstream of transcription start site), promoters, 5’ UTRs, exons, introns, 3’UTRs, and NA (uncategorized). Annotations of repetitive regions were also generated for these piRNAs (b) The repeat annotation was performed by using the R package valr against the repeat annotation downloaded from the UCSC genome browser. Types of repetitive regions were condensed into the following categories: DNA, LINE, Low_complexity, LTR, other, RC/Helitron, satellite, Simple_repeat, SINE, and unknown. Tables S3 (gene annotations) and S4 (repeat analysis) contain the frequency and proportions labelled on the graph for each analysis.

Among the piRNAs potentially altered by Pb exposure (p-value threshold of < 0.05 and |logFC| > 1), we found a higher proportion of piRNAs (from combined sexes, females, and males) mapping to exons (29–36% vs. 4% in a random region) and a lower proportion of piRNAs mapping to introns (20–23% vs. 51% in a random region) compared to those present in a random region of the genome (Figure 5(a)). In piRNAs mapping to repetitive regions, a smaller proportion of piRNAs (from combined sexes, females, and males) mapped to LINEs (7–13% vs. 37% in a random region) and a greater proportion of piRNAs mapped to LTRs (32–39% vs. 25% in a random region) compared to a random region of the genome (Figure 5(b)). Both the combined (16%) and female (16%) showed similar, lower proportions of SINEs compared to random (22%), while males showed a higher proportion of piRNAs mapping to SINES (25%; Figure 5(b)). Tables S3 (gene annotations) and S4 (repeat analysis) contain the frequency and proportions labelled on the graph for Figure 5(a,b), respectively.

### Gene ontology analysis for heart-specific piRNAs sensitive to perinatal Pb exposure

**Figure 6.**
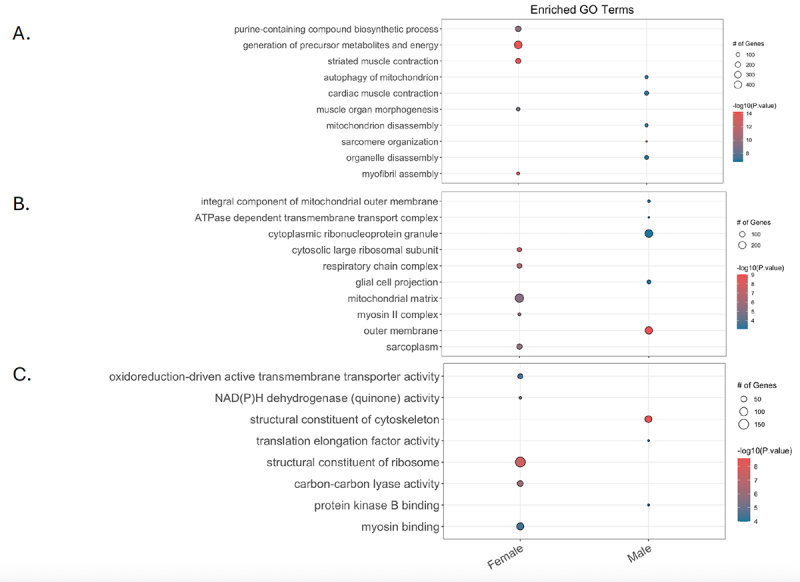
Gene Ontology analysis for piRnas potentially altered by developmental Pb exposure. For combined sex, male, and female analyses, gene Ontology enrichment was carried out using chipenrich in R for the categories of biological processes (GOBP; a), cellular compartment (GOCC; b), and molecular function (GOMF; c) for piRnas found to be potentially altered by Pb exposure (p-value < 0.05 and logFC > 1 or < −1). The top 5 unique GO terms for male and female for all three GO categories are shown here. For all three, GO enrichment was filtered by FDR < 0.05, number of geneset genes is shown by circle size, -log10(p-value) is indicated by red for a more significant value and blue for less significant, and specific GO terms are listed on the left side. Tables S5 (GOBP), S6 (GOCC), and S7 (GOMF) contain the full enrichment data for these analyses.

We performed Gene Ontology overrepresentation analysis for piRNAs potentially altered by Pb exposure (p-value < 0.05 and logFC > ±1) in both sexes combined, females, and males using biological processes (GOBP), cellular compartment (GOCC), and molecular function (GOMF). Figure 6 highlights the biological processes that were significantly differentially expressed (FDR < 0.05) and unique to each sex for piRNAs sensitive to Pb exposure. For GOBP, 48 terms were enriched in the combined analysis, 57 terms were enriched in females, and 39 in males (Table S5). Notably, 52 terms were unique to females (not in males) and 34 were unique to males (Table S5 and Figure 6(a)). For GOCC, 16 terms were enriched for the combined analysis, 23 for females, and 20 for males (Table S6). Of these, 11 terms were unique to females (not in males) and 8 were unique to males (Table S6 and Figure 6(b)). For GOMF, 4 terms were enriched in the combined analysis, 17 in females, and 9 in males (Table S7). Of these, 11 terms were unique to females and 3 were unique to males (Table S7 and Figure 6(c)).

The top 5 GOBP, GOCC, and GOMF terms uniquely enriched for each sex are shown in Figure 6. These include processes related to heart structure and function for both females and males, such as myofibril assembly (FDR 1.26e-11), striated muscle contraction (FDR 1.26e-11), muscle organ morphogenesis (FDR 9.63e-07), myosin II complex (FDR 1.54e-06), sarcoplasm (FDR 2.79e-06), and myosin binding (FDR 4.45e-03) for females and sarcomere organization (FDR 2.17e-09) and cardiac muscle contraction (FDR 5.86e-05) for males.

Additionally, processes related to mitochondria were uniquely enriched in both females and males. For females, these included respiratory chain complex (FDR 2.74e-07), mitochondrial matrix (FDR 6.19e-06), and NAD(P)H dehydrogenase (quinone) activity (FDR 5.06e-04). For males, these included autophagy of mitochondrion (FDR 4.89e-05), mitochondrion disassembly (FDR 4.89e-05), outer membrane (FDR 8.91e-08), and integral component of mitochondrial outer membrane (FDR 1.32e-02) for males.

Finally, processes related to energy metabolism were uniquely enriched for both females and males, including generation of precursor metabolites and energy (FDR 1.26e-11), purine-containing compound biosynthetic process (FDR 1.43e-07), carbon-carbon lyase activity (FDR 1.27e-04), and oxidoreduction-driven active transmembrane transporter activity (FDR 4.45e-03) for females and ATPase-dependent transmembrane transport complex (FDR 3.22e-03) and protein kinase B binding (FDR 1.13e-02) for males.

### Assessment of potential mRNA targets

**Figure 7.**
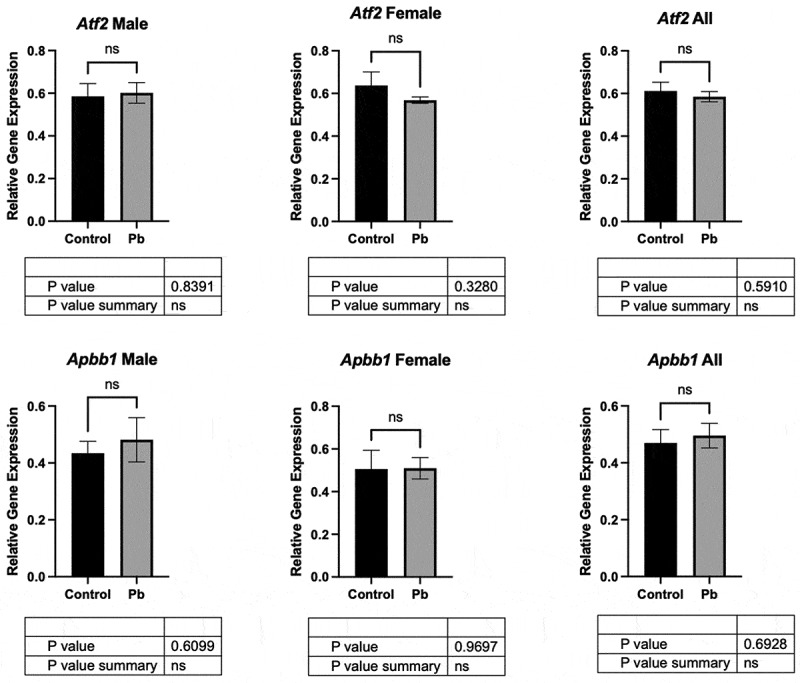
Assessment of potential mRNA targets: mRNA levels (relative gene expression) were assessed for two potential target genes from the same samples in which piRNA analysis was performed. *Atf2* and *Apbb1* were selected from the piRNA mapped targets shown in table S8 for qRT-PCR analysis. Both individual and combined sexes were assessed with no statistical significance for comparison between control and Pb samples. P-values from unpaired t-tests are shown in each comparison for each gene.

Two potential target genes, *Atf2* and *Apbb1*, were selected from the piRNA mapped targets shown in Table S8 for qRT-PCR analysis. We found Pb exposure did not result in significant gene expression changes in sex-specific or combined analysis (Figure 7). P-values from unpaired t-tests are shown in each comparison for each gene.

## Discussion

### Sex-stratified baseline piRNA expression in murine hearts

Notably, this work is the first to identify 18,956 piRNAs through sodium periodate exclusion in the mouse heart and to characterize sex-specific piRNA effects in adult murine offspring upon perinatal Pb exposure (Figure 1). Sex-stratified analysis revealed larger numbers of piRNAs expressed from female mice (9,231) compared to male mice (5,972; Table 1). Of these, 2,436 were found to be uniquely expressed in females and 1,768 unique in males (Figure 2). These expression profiles may indicate that piRNAs play an important role in the heart. Our previous study found around 40,808 enriched peaks (24,777 piRNA meeting length criteria) expressed in mouse testes, and 5,494 enriched peaks (3,147 piRNA meeting length criteria) in the hippocampus (much more than the brain cortex) [15]. Germline piRNAs are well understood to silence transposable element motility, while piRNA function in the hippocampus is still being studied, but it has been suggested they could play roles in brain plasticity and successful aging (proper neurogenesis and maintenance) [50,51]. One study of Piwil2 in adult mouse neural progenitor cells found it was dynamically expressed and essential for proper neurogenesis [51]. Current work by our lab is assessing PIWIL expression and function in differentiation of human iPSC-derived cardiomyocytes to investigate the importance of piRNA and PIWILs in cardiac function.

Another study of piRNAs in human embryonic stem cells undergoing differentiation to cardiomyocytes found 171 piRNAs in cardiac cells mapping to critical biological processes in the heart, using size exclusion for piRNA detection [25]. Although the current study used a different species as well as samples from whole adult hearts rather than cardiac cells, by also using sodium periodate methods rather than size exclusion, the current study represents a more rigorous and robust method of detecting piRNA [25].

### Possible exonic targeting by piRNAs

Both our baseline piRNA map as well as those altered by Pb (p-value < 0.05) showed that piRNAs derived from intronic regions were proportionately expressed at lower levels than expected by chance and exons at higher levels than expected by chance (Figures 3 and 5). Given that the current results indicate a higher proportion of piRNAs map to exons rather than intronic regions, this could point to potential novel mechanisms for both baseline piRNA function in the heart as well as how Pb may alter processes in the heart. In mice, piRNAs have been previously described to play roles in regulation of mRNA in CVD, including regulation of acetylation of transcription factor *Tfec* mRNA involved in cardiomyocyte death after myocardial infarction, suppressing methylation of *Atf7* mRNA to regulate necroptosis in cardiomyocytes, playing a role in advancing cardiac fibrosis through direct inhibition of *Apln* mRNA in cardiac fibroblasts, and regulating cardiac hypertrophy through controlling methylation of *Parp10* mRNA [52–55]. Notably, none of our piRNAs which changed in expression by Pb were found to be mapped to these four specific mRNA targets, although the female data did show expression of piRNA mapping to another transcription factor in the same family as *Atf7*, *Atf2* (Table S8) [53,56]. We hypothesize that piRNAs we discovered to be changed in expression by Pb and mapping to exonic regions could have roles in the development of CVD. We assessed relative gene expression of two potential exonic targets based on our mapping data in our samples: *Atf2* and *Apbb1*, and found no statistical significance for either gene when comparing Pb vs control for female, male, and combined samples (Figure 7).

One important note is that the piRNAs we identified mapping to exonic regions do not necessarily imply a regulatory function. It is possible that the enrichment in exons reflects the presence of degraded mRNA fragments that were misidentified as piRNAs, leading to potential false positives. Future work is needed to determine whether the piRNAs identified here actively regulate mRNA expression and whether their target genes are involved in cardiovascular disease (CVD).

### Pb exposure effects on piRNAs targeting transposable elements

Further, of those piRNAs that originated from intronic regions in control hearts, fewer coincided with LINE expression than expected by chance, and the same effect was observed in the context of Pb exposure (Figures 3(b) and 5(b)). However, relatively similar proportions of piRNAs mapping to LTRs were seen in control hearts compared to chance, while a greater proportion of LTRs compared to chance were seen in Pb exposed samples (Figures 3(b) and 5(b)). While the exact mechanism of piRNAs mapping to these regions is unclear, this could imply that Pb exposure affects the functionality of piRNAs in repressing transposable element motility, a well-characterized function of piRNAs in the germline that is less well understood in somatic tissue [7]. It is important to note that transposable elements are highly repetitive, and our study limited each read to 10–45 nucleotides and 1000 mapping locations [57]. Therefore, it is possible that some mapping sites were missed, particularly for LINEs [58].

### Sex-stratified characteristics of piRNA expression

Around 38% more piRNAs were discovered through analysis of female control hearts compared to males, and there were many uniquely expressed piRNAs in both female (2,436) and male (1,768) baseline mapping (Table 1 and Figure 2). Despite these differences, the genomic annotations for piRNAs from control hearts did not show sex-specific differences for both genome-wide analysis and analysis of repetitive regions (Figure 3). Taken together, this suggests the potential for functional redundancy despite differences in piRNA expression.

In testes and ovaries, the primary function of piRNA is the same (to silence transposon expression in germ cells), however differences in timing and type of expression have been found due to differences in oogenesis and spermatogenesis [59]. Further, mutations in essential piRNA pathway genes have been shown to result in male sterility but have no effect on female fertility in mice [60]. Therefore, because of biological differences in females and males, piRNA in the heart could be expressed differently but have similar functions.

Research is just beginning to uncover how sex-specific epigenetic changes are related to the development of CVD, and previous work has found that Pb alters DNA methylation in a sex-specific manner in the heart [42]. Sequencing of hearts from mice that underwent developmental exposure to Pb revealed a greater number of Pb-altered piRNAs in adult females (847) compared to age-matched adult males (187), and only 23 piRNAs that overlapped between the two analyses (Table 2 and Figure 4). The region of genome piRNAs originated from did not vary greatly based on sex, but the mapping to repetitive regions showed some variation based on sex (Figure 5). Most notably, males showed greater proportions of piRNAs mapping to DNA transposons, LINEs, and SINEs and smaller proportions to low complexity regions and simple repeats (Figure 5). Therefore, Pb exposure may alter piRNA expression in the heart in a sex-specific manner that has functional consequences.

To further investigate this point, Gene Ontology analysis revealed these piRNAs may be related to unique biological processes in males and females. However, these biological processes have overlapping roles related to heart function. Of the top five unique GO terms for GOBP, GOCC, and GOMF for both male and female piRNAs expressed with Pb exposure, processes related to the mitochondria or energy metabolism were enriched (5/13 top pathways for males and 5/15 for females). Given the high energy demands of the heart, mitochondria are critical for normal function, and dysregulation of mitochondrial pathways in the heart are known to be involved in various CVDs including atherosclerosis, ischemia-reperfusion injury, hypertension, diabetes, cardiac hypertrophy, and heart failure mainly through mechanisms of oxidative stress [61]. Pb is known to disrupt energy metabolism and cause oxidative stress, particularly in the brain, but how Pb may affect heart development through oxidative stress is largely unknown [62,63]. Importantly, this GO data suggest that changes in piRNA expression by Pb (p-value < 0.05) may be related to energy metabolism in both sexes, but the specific pathways enriched were distinct in each sex.

Processes related to muscle cell function and structure were enriched in both female (6) and male (2) analyses. For example, myofibril assembly was enriched for females, and sarcomere organization was enriched for females, with sarcomeres functioning as the unit of contraction within a myofibril. Dysregulation of heart muscle function has been implicated in various CVDs, including cardiomyopathies and heart failure [64–66]. Both female and male pathways also included processes related to ribosomal structure and function (2 for both males and females), alterations of which may be related to the development of CVD [67].

Pathways with unique functions were also present for both sexes. In males, protein kinase B (Akt) binding was enriched, which has been shown to play roles in heart failure, atherosclerosis, myocardial infarctions, hypertension, and cardiomyopathies [68]. The mapping of piRNAs potentially dysregulated by Pb to regions that regulate distinct pathways with overlapping functional outcomes highlights the notion that while female and male piRNA may originate from different regions, they may play similar roles, as in the germline. Further studies are needed to examine piRNA function in the female and male heart in relation to the origin of piRNA expression.

### Study limitations

One major limitation of the present study is that smRNA was isolated from whole heart tissue, which may include fibroblasts, blood, and other cell types. Future studies are necessary to define cell type-specific piRNAs in the heart. Another limitation of this study is the limited translatability from mice to humans, as previous work suggests that while piRNA associated proteins like PIWILs and piRNA cluster locations are conserved between species, there are major differences in the actual sequences of piRNAs themselves [11,69,70]. Further, only mice at 5 months of age were used for this study, and to understand fully the effects of developmental Pb exposure, further investigations should look at other time points in the life course, as well as piRNA expressed during cardiac differentiation in *in vitro* models. Additionally, since we used the piRNAs discovered at baseline to assess for expression changes induced by Pb exposure, this limits the detection potential of novel piRNAs induced by Pb exposure.

Future work should also examine the coupling between expression of piRNA and their targets, at baseline and in the context of Pb exposure. A recent paper from our group carried out RNA sequencing and DNA methylation analysis of cardiac genes from the same murine perinatal model of Pb exposure used in this paper, and did not report any significantly differentially expressed genes (DEGs) with Pb exposure at 3 week, 5 month, and 10 month time points [71]. However, this work discovered different temporal gene expression changes in control and Pb exposed hearts that were also unique by sex [71]. This could mean that piRNA differences shown here are reflected by these temporal shifts, and that perhaps we are missing earlier windows in gene expression changes given the crucial roles piRNA play in development. Furthermore, piRNA could be acting in a post-transcriptional manner thereby regulating protein levels rather than mRNA or affecting expression of TEs which are difficult to capture in traditional RNA sequencing due to their repetitive nature. Further work is required to investigate earlier timepoints (perhaps fetal gene expression changes), proteomics, as well as qPCR verification of potentially targeted TEs, such as LTRs which were identified as piRNA targets related to Pb exposure in this work.

## Conclusions

Overall, we characterized sex-stratified piRNA expression in both control and Pb-exposed murine hearts. We found robust expression of piRNA in the heart, many of which are mapped to exonic regions, indicating potential future areas of discovery. In addition to providing a foundation for sex-specific piRNA expression in the heart, these findings suggest a novel epigenetic mechanism by which developmental Pb exposure may impact CVD risk later in life. Interestingly, while piRNA expression differed between females and males, GO analysis revealed that the functional consequences of Pb exposure may be similar. Future studies will link these molecular changes to potential gene or transposable element expression changes as well as Pb-induced alterations in cardiac function.

## Supplementary Material

Supplemental Material

## Data Availability

The data supporting the findings of this study are available within the article and its supplementary materials. Sequencing data has been deposited in the NCBI Sequencing Read Archive (SRA) with the accession number PRJNA1257456 and will be made available upon publication of this manuscript.
