# Supplementary material for: Sex-stratified piRNA expression analysis reveals shared functional impacts of perinatal lead (Pb) exposure in murine hearts
Source: Epigenetics. 2025 Aug 10;20(1):2542879. doi: 10.1080/15592294.2025.2542879 (PMC12341058; doi:10.1080/15592294.2025.2542879)
Supplement: Supplemental Material [file KEPI_A_2542879_SM7906.docx]

**Supplementary Materials**

Supplemental data for this article can be found at the repository here: <https://github.com/kimbopossible/Epigenetics_Murine_piRNA_Pb>
